# A Qualitative Exploration of Multilevel Factors Affecting Sleep Quality Among Minoritized Older Adults

**DOI:** 10.1093/geroni/igaf122.3090

**Published:** 2025-12-31

**Authors:** Mary Janevic, Kimberlydawn Wisdom, Martha Quinn, Daniel Whibley, Philip Cheng, Rebecca Lindsay

**Affiliations:** University of Michigan School of Public Health, Ann Arbor, Michigan, United States; Henry Ford Health, Detroit, Michigan, United States; University of Michigan School of Public Health, Ann Arbor, Michigan, United States; University of Michigan Medical School, Ann Arbor, Michigan, United States; Henry Ford Health, Detroit, Michigan, United States; University of Michigan School of Public Health, Ann Arbor, Michigan, United States

## Abstract

Poor sleep is a risk factor for dementia and other chronic conditions, and disproportionately affects African American and economically marginalized older adults. Drivers of sleep inequities exist at the individual, household/neighborhood, and structural levels. We conducted 21 semi-structured interviews with adults aged 50 to 74, 77% African American, who reported persistent “poor” or “very poor” sleep quality over 2+ months. We sought to identify the multilevel factors that impact sleep and elicit feedback on potential components of a behavioral sleep intervention tailored for this community. Using a rapid qualitative analysis approach, we first developed a template reflecting domains of interest (e.g., factors hindering sleep), and then extracted data from transcripts for each domain. We aggregated and reviewed data for main findings. Results revealed that sleep impediments were multilevel. Individual level factors included feeling worried/anxious and being disturbed by pain. Household/neighborhood factors were needing to be “on alert” at night due to security concerns or to care for elderly parents, noise from adult children or pets, and sleeping in a too-warm room. Most participants did not have difficulty falling asleep but woke up repeatedly and had trouble going back to sleep. Appealing features of an intervention included improving sleep without medications; having regular contact with a community health worker; wearing a watch to track sleep; and resources such as videos and podcasts. Findings suggest that a behavioral sleep intervention tailored to the specific needs of older adults in a marginalized community has the potential to be both acceptable and efficacious.

